# Marginal bone loss around non-submerged implants is associated with salivary microbiome during bone healing

**DOI:** 10.1038/ijos.2017.18

**Published:** 2017-06-16

**Authors:** Xiao-Bo Duan, Ting-Xi Wu, Yu-Chen Guo, Xue-Dong Zhou, Yi-Ling Lei, Xin Xu, An-Chun Mo, Yong-Yue Wang, Quan Yuan

**Affiliations:** 1 State Key Laboratory of Oral Diseases, National Clinical Research Center for Oral Diseases, West China Hospital of Stomatology, Sichuan University, Chengdu, China; 2 School of Dentistry, University of California, Los Angeles, CA, USA; 3 Department of Operative Dentistry and Endodontics, West China Hospital of Stomatology, Sichuan University, Chengdu, China; 4 Department of Oral Implantology, West China Hospital of Stomatology, Sichuan University, Chengdu, China

**Keywords:** dental implant, illumina sequencing, marginal bone loss, oral microbiome, peri-implantitis

## Abstract

Marginal bone loss during bone healing exists around non-submerged dental implants. The aim of this study was to identify the relationship between different degrees of marginal bone loss during bone healing and the salivary microbiome. One hundred patients were recruited, and marginal bone loss around their implants was measured using cone beam computed tomography during a 3-month healing period. The patients were divided into three groups according to the severity of marginal bone loss. Saliva samples were collected from all subjected and were analysed using 16S MiSeq sequencing. Although the overall structure of the microbial community was not dramatically altered, the relative abundance of several taxonomic groups noticeably changed. The abundance of species in the phyla *Spirochaeta* and *Synergistetes* increased significantly as the bone loss became more severe. Species within the genus *Treponema* also exhibited increased abundance, whereas *Veillonella*, *Haemophilus* and *Leptotrichia* exhibited reduced abundances, in groups with more bone loss. *Porphyromonasgingivalis*, *Treponemadenticola* and *Streptococcus intermedius* were significantly more abundant in the moderate group and/or severe group. The severity of marginal bone loss around the non-submerged implant was associated with dissimilar taxonomic compositions. An increased severity of marginal bone loss was related to increased proportions of periodontal pathogenic species. These data suggest a potential role of microbes in the progression of marginal bone loss during bone healing.

## Introduction

Dental implantation has become a principal, established therapy to restore missing natural teeth in regular clinical practice. The rehabilitation technique based on dental implantation can provide a wide variety of treatment options to patients given its high predictability and survival rate, but implants are not completely free of complications and failure. Marginal bone loss (MBL) around dental implants is a serious problem,^1, 2^ and extensive bone loss has long been regarded as one key factor contributing to implant failure.^3, 4^ Since the 1980s, MBL assessment with intra-oral radiographs has been regarded as a critical criterion to assess implant success.^5^ The accepted criteria for implant success are defined as 1–1.5 mm of bone loss during the first year after loading and <0.2 mm annually thereafter.^6, 7^

MBL, which occurs during the bone-healing period for two-stage implants, exists around non-submerged dental implants and may represent a significant threat to implant longevity. According to the literature, both biological and biomechanical factors may be related to MBL during bone healing. Host-related factors include plaque control,^8^ smoking^9^ and wound-healing capacity.^10, 11^ Implant design characteristics related to MBL may involve platform switching,^12^ the implant surface^13^ and neck microthreads.^14^ Furthermore, other contributing factors, such as surgical trauma^15^ and different restorative protocols,^16^ may also play a role in this process.

Microbiological studies have demonstrated that the biofilm associated with peri-implantitis or implant failure differs substantially from that associated with healthy implants.^17, 18, 19^ Furthermore, interactions among physicochemical surfaces, bacteria and the host immune system are important aspects for determining peri-implant bone loss and the long-term stability of an implant.^20^ These findings inspired the further exploration of the association between oral bacteria and MBL during the bone-healing period. Only limited studies have focused on this relationship.

Notably, almost all previous studies focused on the microenvironment around implants. However, the mouth is an open system with continual flow of liquid contacting the surfaces of both hard and soft tissues. Saliva harbours numerous and diverse microorganisms, thus acting as an intermediary for transmitting and dispersing these microorganisms intraorally.^21^ The salivary microbiome has potential connections with the host’s health status, and has promise as a surrogate indicator for health monitoring and disease diagnosis.^22, 23^ However, no comprehensive study has focused on the correlation between MBL during the bone-healing period and the salivary microbiome, to the best of our knowledge.

Hence, in this study, we employed Illumina MiSeq sequencing to investigate the salivary microbiome associated with MBL before stage-II implant surgery. All subjects were divided into three groups (normal, moderate and severe) according to their MBL severities. The working hypothesis was that overall structures and compositions of the three salivary communities are highly correlated with key differences. We hypothesised that individuals with some specific differences in the salivary microbiota may be more prone to MBL during bone healing and that bacteria are significantly associated with MBL progression.

## Materials and methods

### Ethics statement

Participants provided informed consent *via* a signed statement before participation. The human subject protocol was approved by the Institution Review Board of the West China Hospital of Stomatology, Sichuan University (authorisation number WCHSIRB-ST-2016-072).

### Study population

Participants were recruited from individuals seeking care at the Implant Center of West China Hospital of Stomatology. The following inclusion criteria were used: Chinese, >18 years of age, non-smokers in good general healthy, single tissue-level implant with an sandblasted large-grit acid-etched surface (Straumann AG, Basel, Switzerland) in the posterior mandible jaw, and edentulous area and adjacent teeth with healthy periodontal status. The following exclusion criteria were used: antibiotic therapy or oral prophylactic procedures within the preceding 3 months, a need for antibiotic coverage before dental treatment, fewer than 20 teeth present in the dentition; immediate implant or early implant placement, and a need for osseous grafting or other augmentation procedures. All surgical procedures followed the manufacturer’s guidelines of Straumann tissue-level implants. All surgical personnel involved in the treatment of these patients had adequate training in advanced implantology.

### Clinical examination

All subjects were examined by one trained and calibrated examiner preoperatively and 3 months postoperatively (T_0_ and T_3_). Plaque indices and the gingival index were measured at four sites per tooth (mesio-buccal, buccal, disto-buccal and lingual). Probe depth (mm) was measured at six sites per teeth (mesio-buccal, buccal, disto-buccal, disto-lingual, lingual and mesio-lingual) using a standard periodontal probe.

### Marginal bone-level measurement

Patients were examined with the same cone beam computed tomography device (3D Accuitomo 170 cone beam computed tomography (CBCT) device, J Morita, Tokyo, Japan) immediately after surgery and 3 months postoperatively. MBL was measured as follows: the implant platform (the horizontal interface between the implant and the abutment) was used as the reference point. Vertical distances from the reference point to the most coronal level of bone-to-implant contact at both the mesial and distal sites were measured at two time points, respectively;^24, 25^ MBL was measured by subtracting the obtained data from each site. The higher value was used to group the patients. Analyses of radiographs were performed by the same investigator who was blinded and unrelated to the study, and all the evaluations were repeated on a separate occasion within a 6-week interval.

### Sample collection and DNA isolation

Saliva was collected before surgery according to the techniques described by Navazesh.^26^ Briefly, 3~5 mL spontaneous, whole unstimulated saliva was collected from each subject. Volunteers were instructed to refrain from drinking and eating for at least 2 h before sampling and from using oral hygiene products for 12 h before sampling. All samples were stored at −80 °C before further processing. Total DNA was extracted using the QIAamp DNA micro Kit (QIAGEN Sciences, MD, USA) with an extra lysozyme treatment step for lysing the bacterial cells.

### Sequencing and data analysis

The V4 regions of the bacterial 16S rRNA gene were amplified using polymerase chain reaction (95 °C for 3 min; 27 cycles at 95 °C for 30 s, 55 °C for 30 s, 72 °C for 45 s; and a final extension at 72 °C for 10 min). The primers 515F 5′-GTGCCAGCMGCCGCGG-3′ and 907R 5′-CCGTCAATTCMTTTRAGTTT-3′ were used. Purified amplicons were pooled in equimolar ratios and paired-end sequenced (2 × 250) on an Illumina MiSeq platform (Illumina, San Diego, CA, USA) according to standard protocols. The raw reads were deposited into the NCBI sequence read archive database. Raw fastq files were demultiplexed and quality-filtered using QIIME (version 1.9.1). Operational taxonomical units (OTUs) were clustered with 97% similarity cutoff using UPARSE (version 7.1), and chimeric sequences were identified and removed using chimaera checking (UCHIME). The taxonomy of each 16S rRNA gene sequence was analysed by RDP Classifier (http://rdp.cme.msu.edu/) against the Silva (SSU123) 16S rRNA database using confidence threshold of 70%.^27^ Alpha-diversity indices were estimated from the number of observed OTUs, Chao1 and the Shannon diversity index, which reveals both species richness and evenness. Non-metric multidimensional scaling (NMDS) ordination was used to determine the degree of dissimilarity between pairs of bacterial communities using the Bray–Curtis distance method. Two different nonparametric analyses were also employed to examine community differences, including analysis of similarities (ANOSIM) and nonparametric multivariate analysis of variance (adonis).

### Statistical analysis

Participants were grouped into the following three categories according to the degree of MBL found around their dental implants: (i) severe group (*n*=36): MBL≥1 mm; (ii) moderate group (*n*=36): 0.5 mm≤MBL<1 mm; and (iii) normal group (*n*=28): MBL<0.5 mm. The relative abundances of each bacterial taxon were calculated and are typically presented as the mean±standard error of the mean (s.e.m.). For multiple comparisons between the three different groups, ANOVA (one-way) and *post hoc* least significant difference (LSD) was performed. The significance threshold was set at 0.05.

## Results

### Patients’ clinical characteristics and overall sequence statistics

One hundred volunteers were selected for this study (50 men and 50 women; mean age: 46.78 years; range: 18–60 years; all of Han nationality). Clinical metrics for the participants are detailed in Table 1. No significant differences in patients’ characteristics were observed among the three groups and between the two time points. In addition, no changes were reported in terms of living habits or health conditions during the bone-healing period.

Sequencing produced a total of 4 356 945 raw 16S rRNA sequences with an average length of 395 bp. After preprocessing, 28 known phyla and 489 genera were identified. A total of 994 OTUs were detected at 3% dissimilarity using the Uclust programme.^28^ In total, 220 of these were singletons and were excluded from further statistical analysis.

### Comparison of the phylogenetic composition among the three salivary communities

At the phylum level, the microbial compositions of the three communities were similar (Table 2), and the vast majority of sequences (>95%) belonged to one of five phyla: *Firmicutes*, *Proteobacteria*, *Bacteroidetes*, *Actinobacteria* and *Fusobacteria*. The phyla *Spirochaeta* (*P*<0.05) and *Synergistetes* (*P*<0.05) were highly associated with the moderate and severe groups, and *TM7* (*Candidatus Saccharibacteria*) and *Tenericutes* were also more abundant in those two groups than in the normal group. Among all the three groups, the five predominant phyla were present at similar levels.

**Figure 1:**
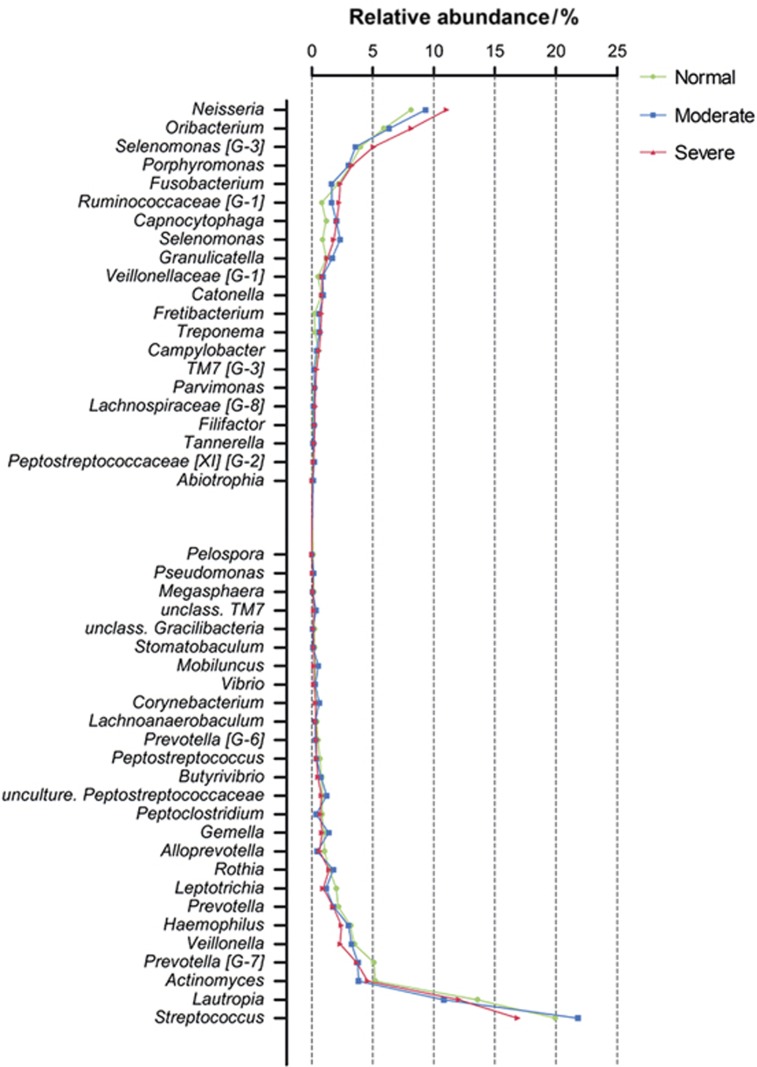
**Microbial differences among the normal, moderate and severe groups at the genus level.** The graph presents levels for the genera for which the abundances were ≥0.1% different between groups. The taxa were sorted according to the magnitude of change.

**Figure 2:**
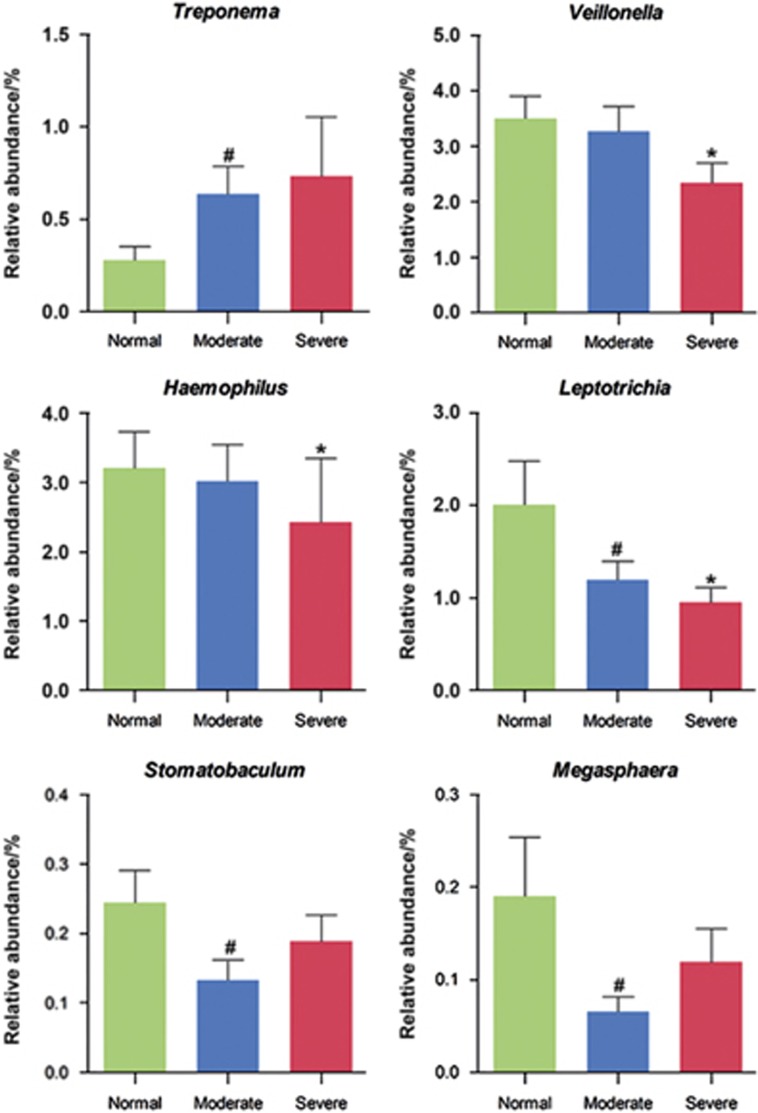
**Genera for which the abundances significantly differed between the groups.** ANOVA (one-way) and *post hoc* least significant difference (LSD) were performed for multiple comparisons between the three different groups. *P<0.05 between severe group and normal group; ^#^*P*<0.05 between moderate group and normal group.

We then investigated the microbial shift in more detail at the genus level. A genus-level phylogenetic tree constructed using MEGA 5 is presented in Supplementary Figure S1. The overall abundance and the magnitude of the difference across the normal, moderate and severe groups are indicated by the bars. *Streptococcus*, *Lautropia*, *Neisseria*, *Oribacterium*, *Actinomyces*, *Prevotella [G-7]* and *Selenomonas [G-3]* were the predominant genera. With the exception of the genera of *Actinomyces*, *Prevotella [G-7]* and *Selenomonas [G-3]*, the abundances of which were slightly altered, the top seven genera remained almost the same as severity increased from normal to severe. Figure 1 presents genera for which the abundance differed by ≥0.1% between the groups. Figure 2 presents the genera for which the abundance significantly differed among the three groups. Compared with the normal group, the relative abundance of species in the genus *Treponema* was significantly increased (*P*<0.05) in the moderate group. *Neisseria*, *Oribacterium, Selenomonas [G-3]*and *Capnocytophaga* were more abundant in the normal group than in the severe group. In contrast, the reverse trend was noted for *Haemophilus*, *Leptotrichia* and *Veillonella*, which were significantly (*P*<0.05) more abundant in the normal group.

The differences in bacterial composition among the three groups were also reflected by the Venn diagram of shared and different OTUs (Supplementary Figure S2). In total, 54.9% of the total OTUs detected (774, with the exception of singletons) were shared among all three communities. The numbers of OTUs exclusively detected in the severe, moderate and normal groups were 54, 66 and 35, respectively. Twenty-two OTUs were shared between the severe and normal groups, and 27 OTUs were shared between the moderate and normal groups. However, more OTUs (66) were shared between the moderate and severe groups.

In addition, we evaluated the OTUs that were present in two or more samples with a mean relative abundance of >0.01% (Supplementary Table S2). In total, 29 of the 250 health-associated OTUs were significantly different among the three groups (*P*<0.05). Notably, most of those OTUs demonstrating statistically increased abundances in the severe group were Gram-negative anaerobic taxa.

### Comparison of the phylogenetic structure among the three salivary communities

**Figure 3:**
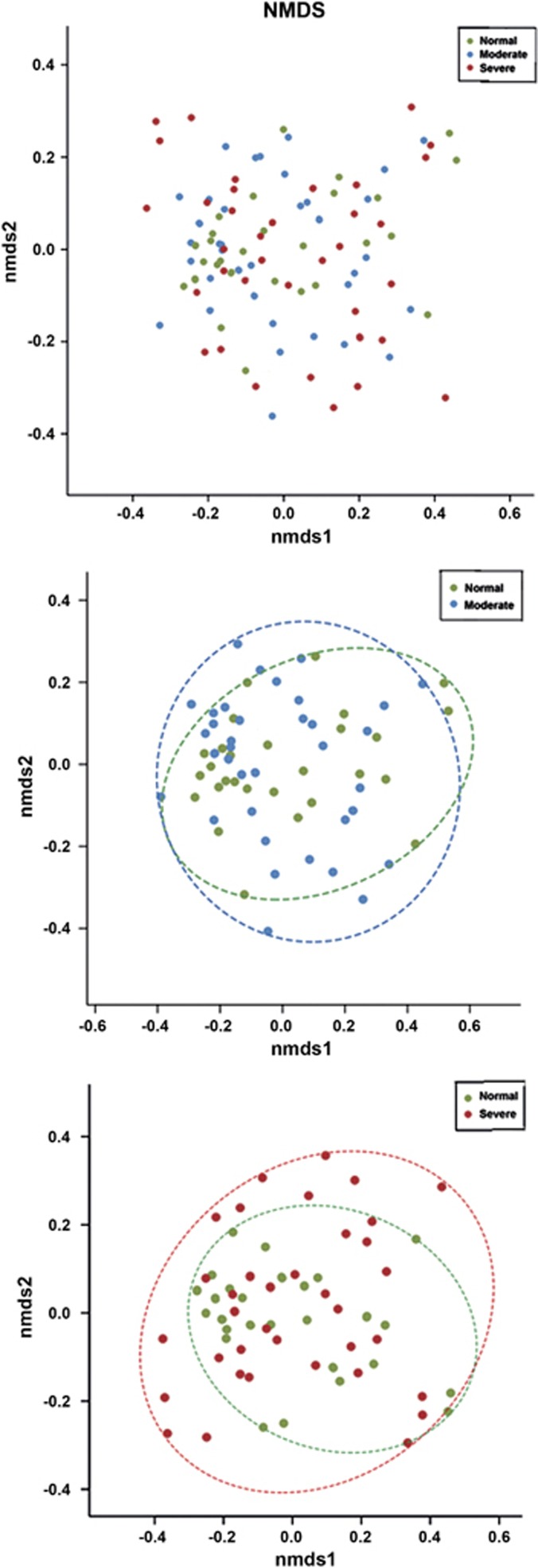
**NMDS analysis.** NMDS based on the Bray–Curtis distance among all the three groups, and between the normal group and moderate or severe groups.

We visualised the differences in the phylogenetic structures among the groups by performing a NMDS ordination. Each data point represents one sample, and the spatial distance between points in the plot is interpreted as the relative difference in the composition of substrate marking. NMDS for these taxa failed to indicate an obvious separation among normal, moderate and severe subjects. The results reveal slight tight clustering of normal samples, but a broader variation in the moderate and severe samples (Figure 3). This difference was more obvious between the severe and normal groups than between the moderate and normal groups. In addition, dissimilarity tests (Adonis and ANOSIM) among the groups also revealed the same trend; however, the differences were not statistically significant (*P*>0.05, Supplementary Table S1). We also examined the differences using the observed OTUs, the Chao index and the Shannon index, and found no significant trends. Oral microbial diversity and richness were similar among the three groups (Supplementary Figure S3).

### Changes in the core microbiota across each community

**Figure 4:**
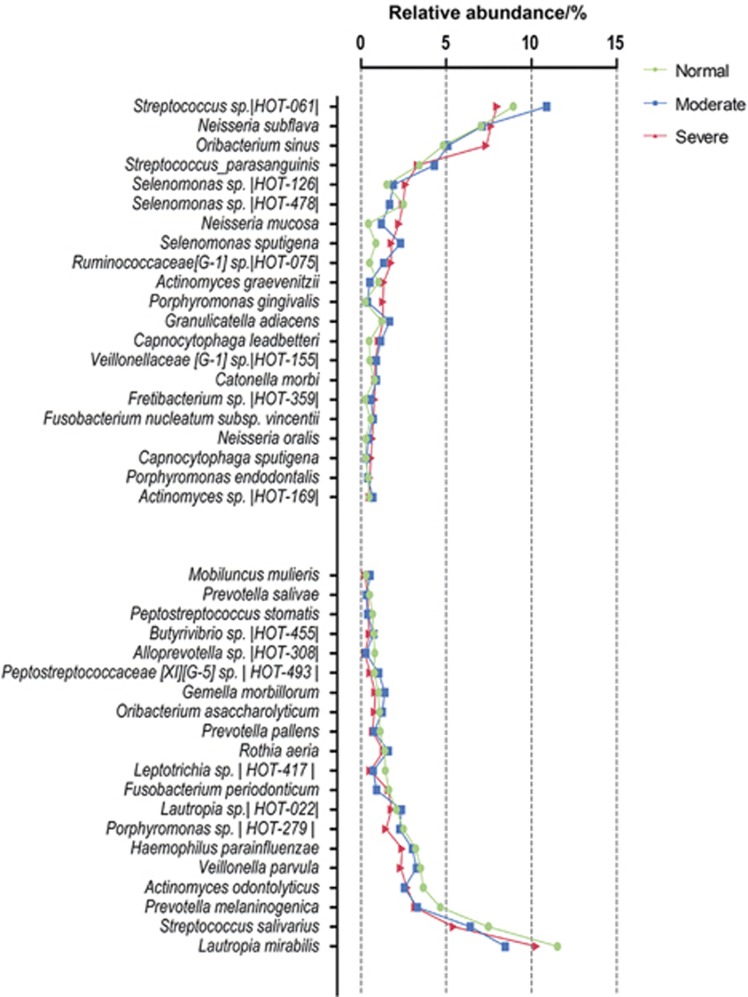
**Microbial differences among the normal, moderate and severe groups at the species level.** The graph presents the most abundant species (≥0.5% abundance) in the normal, moderate and severe samples. The taxa were sorted according to the magnitude of change. The species name or human oral taxon ID in the human oral microbiome is presented.

**Figure 5:**
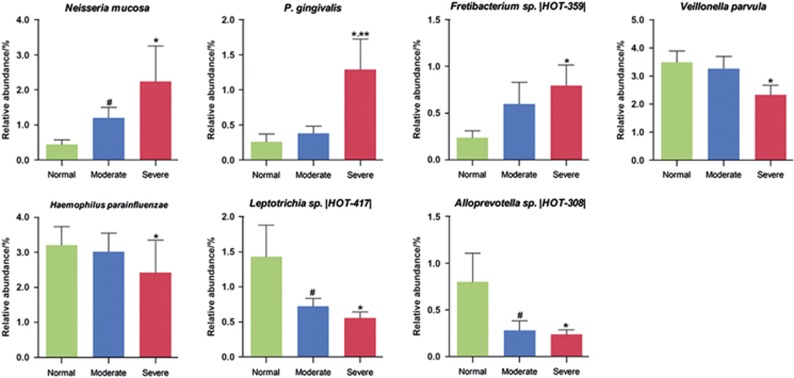
**Species for which the abundances significantly differed between the groups.** ANOVA (one-way) and *post hoc* least significant difference (LSD) were performed for multiple comparisons between the three different groups. *P<0.05 between severe group and normal group; **P<0.05 between severe group and moderate group; ^#^*P*<0.05 between moderate group and normal group.

We examined the core microbiota communities found in peri-implantitis as identified in previous studies.^29,30^ We investigated the most abundant species (>0.5% abundance) among the various communities (Figure 4). Among the total of 41 species, 28 were common to three communities. The numbers of species unique to each community from the normal, moderate and severe groups were three, two and four, respectively. Three species (*Neisseria mucosa*, *Capnocytophaga leadbetteri* and *Fretibacterium* sp. *|HOT_359|*) were identified in both the moderate and severe groups but not in the normal group. One species was shared by the normal and moderate groups. However, no species were shared by the normal and severe groups. These results indicated that although the three communities had the similar overall phylogenetic compositions, some specific species differed significantly during the shift from the normal setting to a disease state (Figure 5).

### Species associated with disease

Considering that MBL was caused by intra-individual microbial infections, we expected that we would observe species that were common to MBL in periodontitis and peri-implantitis. We evaluated the species that are the important periodontal pathogens belonging to the “red complex”, “orange complex” and “yellow complex”, as well as the species having closely relationship with these complexes^31, 32^ (Table 3). We found that *P. gingivalis* (*P*<0.05) and *Treponemadenticola* (*P*<0.05), which are important periodontal pathogens of the “red complex”, were abundant and prevalent in the moderate and severe groups. The moderate and severe groups also appeared to have higher levels of *Streptococcus intermedius* (*P*<0.05), which belongs to the “yellow complex”, than did the normal group.

## Discussion

Research on peri-implant diseases has primarily focused on the late disease stages. However, peri-implant tissue destruction sometimes occurs, and little is known about the how this process is initiated. We are aware of factors that influence MBL during bone healing, but there is a paucity of knowledge regarding the role of bacteria in the progression of this common complication. In this pilot study, we first investigated the complexity of oral microbial communities in patients with different levels of MBL around dental implant during bone healing using MiSeq sequencing. Our results demonstrated the differences between communities at three levels of MBL severity and the correlations of these communities with MBL during bone healing.

Saliva is formed by the mixing of liquid products of the salivary glands, including components of gingival crevicular fluid, serum, bacteria and their products, viruses, fungi, peeled epithelial cells and food particles. Saliva is responsible for maintaining the integrity of the oral cavity. Over the past decade, a number of studies have used saliva samples as an easy, inexpensive and non-invasive diagnostic tool to assess healthy and disease conditions.^33, 34, 35^ Saliva can be considered as a mirror of body health given its association with various oral and systemic conditions, including caries, cardiovascular disease and obesity.^36, 37^ The salivary microbiome, which is specific to each person, exhibits long-term stability on the scale of years.^38^ Inspired by these findings, we collected saliva samples from 100 individuals who were divided into three categories on the basis of MBL severity.

NMDS analysis revealed a tendency for clustering with a narrower distribution of samples in the normal group. Dissimilarity tests revealed “S *vs* N” had the highest *R*-value among the three comparisons. These results indicate that the difference between the severe and normal groups was more obvious than that between moderate and normal groups. This finding may indicate that MBL during bone healing is a complication associated with endogenous bacteria. Rather than being caused by specific pathogens, it is more likely caused by an alteration in the densities of commensal oral bacteria.

Among the three groups, five major phyla constituted the predominant salivary microbiome (namely, *Firmicutes*, *Proteobacteria*, *Bacteroidetes*, *Actinobacteria* and *Fusobacteria*). This finding is consistent with a previous survey on the “core microbiome” of the oral microbial community.^39^ Significant differences were identified among the three groups. The relative abundances of the phyla *Spirochaeta* and *Synergistetes* increased as MBL severity increased. A similar trend was noted for the phylum *TM7*, although it was not statistically significant. Both *Spirochaeta* and *Synergistetes* are Gram-negative anaerobic taxa. Salivary glycoproteins selectively adhere to the abutment or other surfaces of an implant to form the salivary pellicle, where oral bacteria then attach by adhering to epitopes in the pellicle.^40^ In general, the primary colonisers tend to be Gram-positive aerobes and facultative anaerobes,^41^ whereas Gram-negative and anaerobic species are found at increased abundances in mature plaques.^42^ Bone loss around an implant can be stimulated by bacteria, and bone loss subsequently stimulates an anaerobic environment. Together, these conditions become the primary cause of continued bone loss.^43^ Early studies have demonstrated that the subgingival biofilms around failing implants have similar compositions that are characterised by a high proportion of Gram-negative anaerobic rods.^29, 44^ The increased abundances of species in these phyla were in consistent with the previous studies that reported evidence that they were newly identified taxa associated with periodontitis.^45, 46, 47, 48^ The phylum *TM7* also exhibits an association with peri-implant diseases.^30^

Increased MBL was positively related at the genus level to the proportion of the genus *Treponema* that belongs to the phylum *Spirochaeta*. The normal group demonstrated increases in the relative abundances of species in the genera *Veillonella*, *Haemophilus* and *Leptotrichia*. *Veillonella* species interact with *Streptococcus* species during biofilm formation^49^ and thus are likely associated with implant health. Nevertheless, Griffen *et al.*^30^ suggested that *Veillonella* had a positive association with periodontitis. *Haemophilus* species are Gram-negative facultative bacilli, and *H. influenza* is detected in higher amounts in peri-implant lesions.^50, 51^ In the present study, this bacterium was not identified in any of the samples. Many of the species present were unclassified taxa except *Haemophilus parainfluenzae*. Further work is needed to investigate the association between *Haemophilus* species and destructive peri-implant infection. A previous study also reported a similar trend, showing that the peri-implantitis-associated community had a significantly lower level of the genus *Leptotrichia*.^19^ In addition, the abundance of the Gram-positive facultative coccus *Actinomyces* decreased as severity increased, which is consistent with the findings of a previous study.^52^

Notably, we found that *P. gingivalis* and *T. denticola* were significantly abundant in the moderate and/or severe groups. The proportions of the pathogens from the red complex were elevated as severity increased. Members of red complex of periodontal pathogens were previously demonstrated to be the most important microbiota for the progression of periodontitis^31^ and to be positively associated with peri-implantitis.^17, 29, 51^ The relative abundance of *P. gingivalis*, a proposed keystone organism in chronic periodontitis, increased significantly as the severity increased from normal to severe. In addition, its prevalence rates also increased consistently (71%, 75% and 92%, respectively). These results indicate that *P. gingivalis* might act as a predictor of MBL. Furthermore, *P. gingivalis* manipulates immunity *via* a number of mechanisms,^53^ and has been postulated to suppress inflammasome activation by both its immunostimulatory activity and pathogenic synergy with other periodontal bacteria.^54^ Recent studies have also revealed its relationship with systemic and oral health.^55, 56^

The abundance of *Streptococcus intermedius*, which belongs to the yellow complex, exhibited a significantly positive correlation with the severity of MBL during bone healing. This finding is consistent with previous studies suggesting that it is associated with periodontitis.^48, 57^ *Streptococcus sanguinis*, which also belongs to the yellow complex, exhibited a slightly increased abundance in the moderate and severe groups. *S. sanguinis* is a primary coloniser of oral plaques and has been found to be associated with dental implants.^58, 59^

A very interesting finding is that *Neisseria mucosa* was only found in the moderate and severe groups when analysed at the level of most abundant species. *N. mucosa* is a pathogenic bacterium that is a rare but serious cause of endocarditis.^60^ The correlation between it with MBL merits future exploration.

To investigate the relationship between MBL during bone healing and the salivary microbiome in a clinical setting, we recruited patients who exhibited no significant differences in clinical metrics among the three groups at baseline and 3 months after surgery. In addition, no significant differences were noted between the two time points. We also controlled other possible factors that may influence MBL: smoking habits, systemic health and characteristics of the implants.^61, 62^ However, this present study is only a pilot study to investigate the correlation between the MBL during bone healing and the salivary microbiome, and many interactions between species or between species and oral health remain unknown. Further studies are needed to determine the roles of species that are associated with oral health. To shed more light on clinical mechanisms and disease progression, prospectively designed studies that rigorously control confounding factors and have long-term follow-ups (e.g., 1 year after implant surgery), and multicentre clinical trials, are required in the future.

In conclusion, the current study demonstrates that the severity of peri-implant MBL during bone healing is correlated with different salivary microbiomes. This finding suggests a role for the oral microbiota in the onset/progression of MBL. Our findings could provide the basis for further work exploring the bacteria suspected of being associated with MBL in the future. The identification of specific bacterial species that are involved in the progression of peri-implantitis could also provide potential targets for a better prevention programme for MBL during the bone-healing period.

## Figures and Tables

**Table 1:** Demographics and clinical parameters of all subjects

	Groups					
	Normal (*n*=28)		Moderate (*n*=36)		Severe (*n*=36)	
Characteristics	T_0_	T_3_	T_0_	T_3_	T_0_	T_3_
Male/Female	12/16	—	16/20	—	18/18	—
Age (mean±s.d.)	42.0±14.6	—	45.0±14.1	—	52.3±15.9	—
Alcohol drinking	0	0	0	0	0	0
Number of teeth (mean±s.d.)	28.05±2.25	—	28.42±1.77	—	27.34±2.58	—
Plaque index (mean±s.d.)	1.21±0.61	1.20±0.60	1.39±0.60	1.39±0.54	1.44±0.69	1.42±0.68
Gingival index (mean±s.d.)	1.08±0.54	1.06±0.52	1.28±0.50	1.24±0.51	1.40±0.50	1.35±0.54
Probing depth, mm (mean±s.d.)	2.02±0.73	2.01±0.69	2.25±0.60	2.20±0.54	2.51±1.44	2.33±1.39
MBL, mm (mean±s.d.)	0.28±0.13		0.71±0.13		1.55±0.53	

T_0_: baseline (recruitment).

T_3_: 3 months after implant surgery.

s.d., standard deviation.

**Table 2:** The relative abundance (mean±s.e.m.) of the bacterial phylum among the normal, moderate and severe groups

	Groups				Intergroup
Phyla	Normal (*n*=28)	Moderate (*n*=36)	Severe (*n*=36)	*P* value	*P*<0.05
*Firmicutes*	44.69±3.21	49.52±2.64	45.88±2.93	0.478	—
*Proteobacteria*	27.66±3.71	25.66±3.13	27.63±3.57	0.893	—
*Bacteroidetes*	14.01±1.75	12.31±1.67	13.30±1.72	0.790	—
*Actinobacteria*	7.80±1.28	7.25±0.74	7.02±0.98	0.861	—
*Fusobacteria*	4.20±0.63	2.85±0.39	3.29±0.59	0.225	—
*Spirochaetae*	0.29±0.07	0.69±0.12	0.82±0.32	0.046	M *vs* N
*Synergistetes*	0.25±0.08	0.60±0.16	0.81±0.12	0.035	S *vs* N
*TM7*	0.43±0.15	0.54±0.15	0.60±0.28	0.864	—
The others	0.67±0.18	0.60±0.12	0.66±0.12	0.739	—

M, moderate group; N, normal group; S, severe group; s.e.m., standard error of the mean.

ANOVA (one-way) and *post hoc* least significant difference (LSD) were performed for multiple comparisons between the three different niches. The significance threshold was set at 0.05.

**Table 3:** The relative abundance (mean±s.e.m.) of the species associated with disease among the normal, moderate and severe groups

	Groups				Intergroup
Species	Normal (*n*=28)	Moderate (*n*=36)	Severe (*n*=36)	*P* value	*P*<0.05
		Red complex			
*Porphyromonas gingivali*	0.25±0.12	0.38±0.11	1.29±0.43	0.020	S *vs* N; S *vs* M
*Tannerella forsythia*	0.13±0.04	0.12±0.02	0.20±0.04	0.223	—
*Treponema denticola*	0.03±0.01	0.05±0.01	0.09±0.01	0.046	M *vs* N
*Eubacterium nodatum*	0.03±0.01	0.07±0.02	0.07±0.03	0.349	—
*Treponema socranskii*	0.03±0.01	0.06±0.02	0.09±0.05	0.441	—
					
		Orange complex			
*Prevotella melaninogenica*	4.65±0.90	3.31±0.84	3.18±0.76	0.420	—
*Prevotella intermedia*	0.41±0.10	0.34±0.09	0.47±0.16	0.757	—
*Prevotella nigrescens*	0.05±0.02	0.06±0.02	0.05±0.03	0.969	—
*Fusobacterium nucleatum* subsp. *vincentii*	0.57±0.12	0.72±0.15	0.74±0.15	0.688	—
*Fusobacterium periodonticum*	1.62±0.29	0.92±0.16	1.60±0.40	0.164	—
					
		Yellow complex			
*Streptococcus sanguinis*	1.42±0.28	1.97±0.39	1.57±0.43	0.593	—
*Streptococcus intermedius*	0.12±0.03	0.28±0.06	0.42±0.07	0.045	S *vs* N; M *vs* N

M, moderate group; N, normal group; S, severe group; s.e.m., standard error of the mean.

ANOVA (one-way) and *post hoc* least significant difference (LSD) were performed for multiple comparisons between the three different groups. The significance threshold was set at 0.05.
