# Supplementary figures and images for: Marginal bone loss around non-submerged implants is associated with salivary microbiome during bone healing
Source: Int J Oral Sci. 2017 Jun 16;9(2):95–103. doi: 10.1038/ijos.2017.18 (PMC5518974; doi:10.1038/ijos.2017.18)

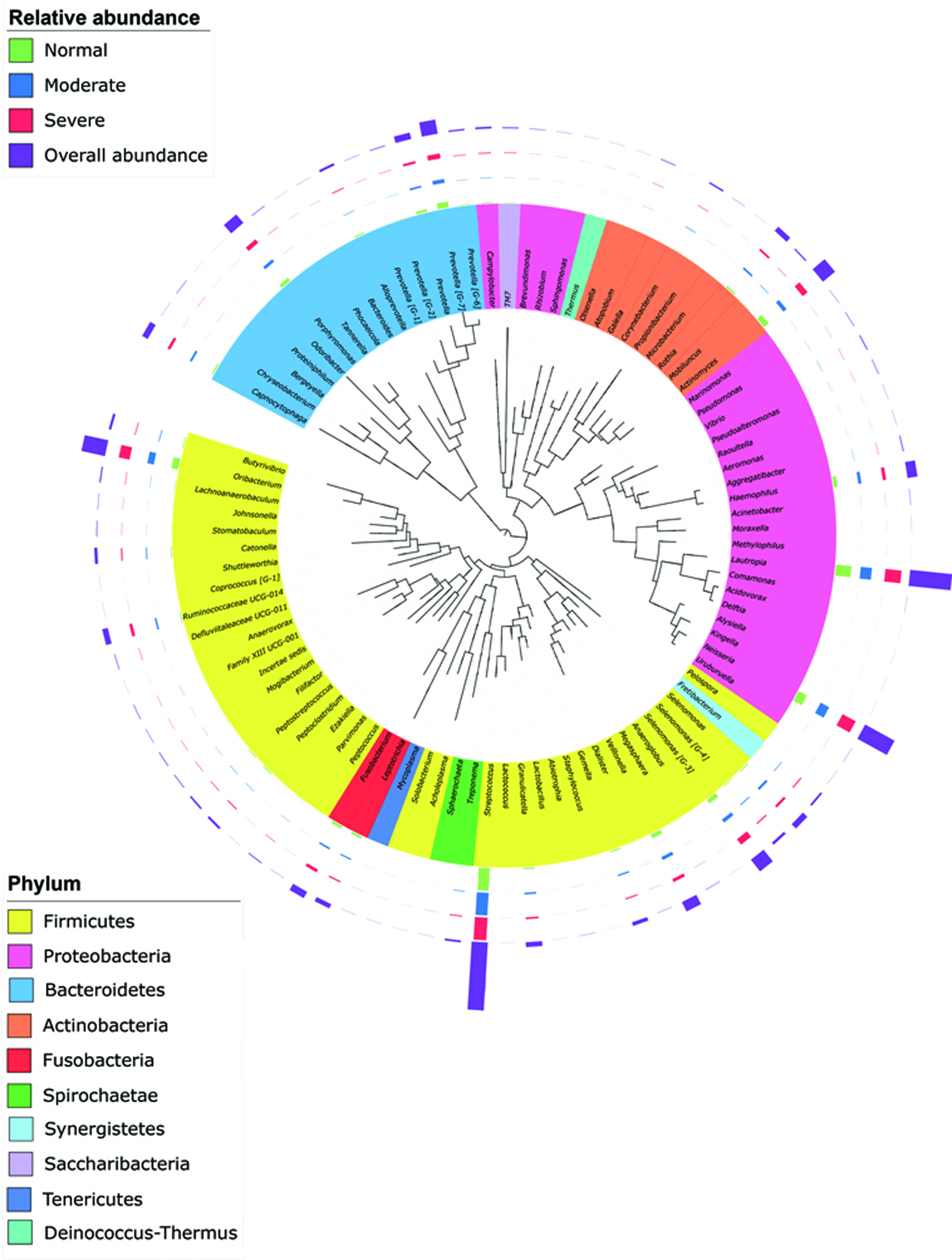

Supplement: Supplementary Figure S1 [file ijos201718x1.tif]

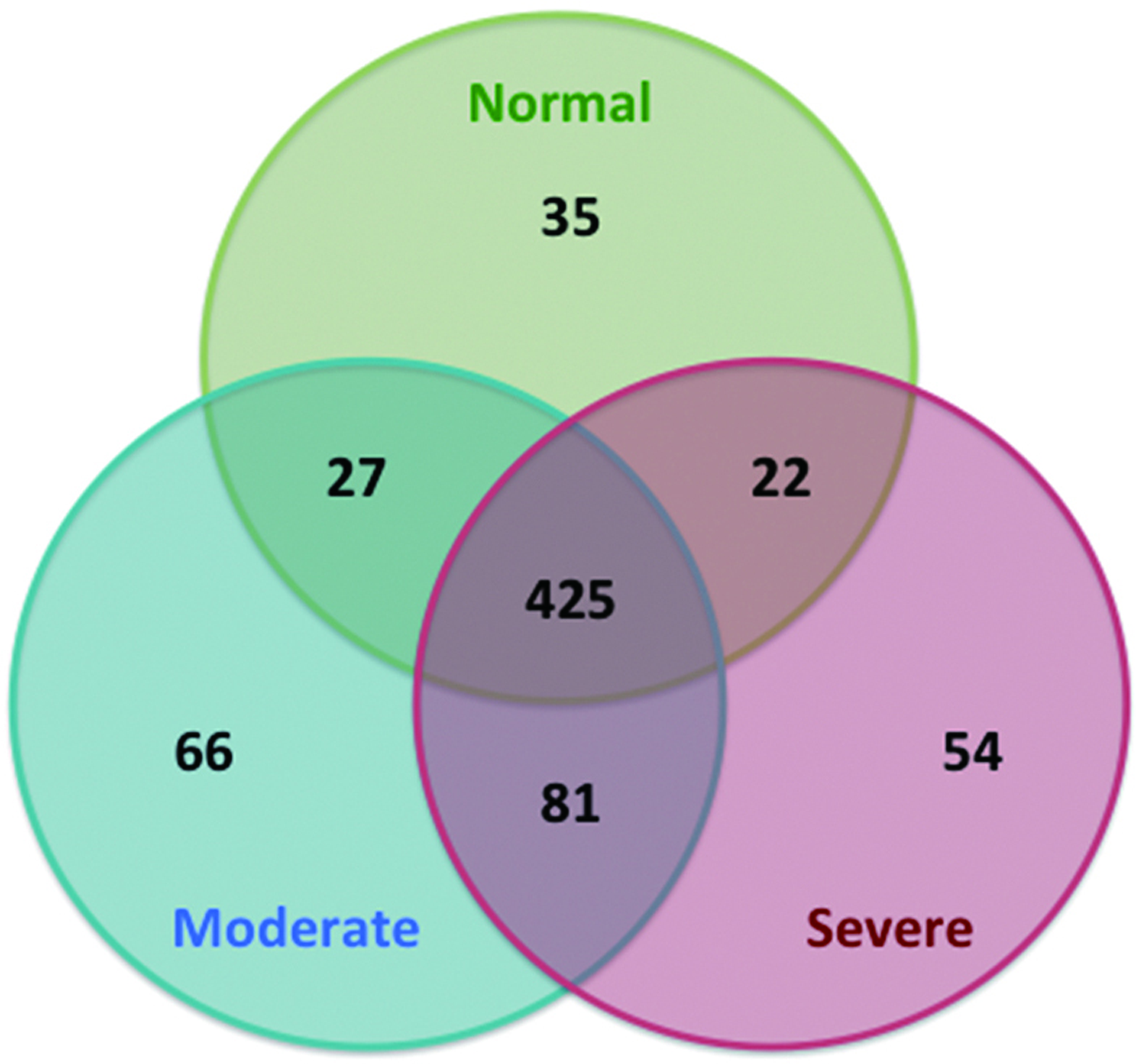

Supplement: Supplementary Figure S2 [file ijos201718x2.tif]

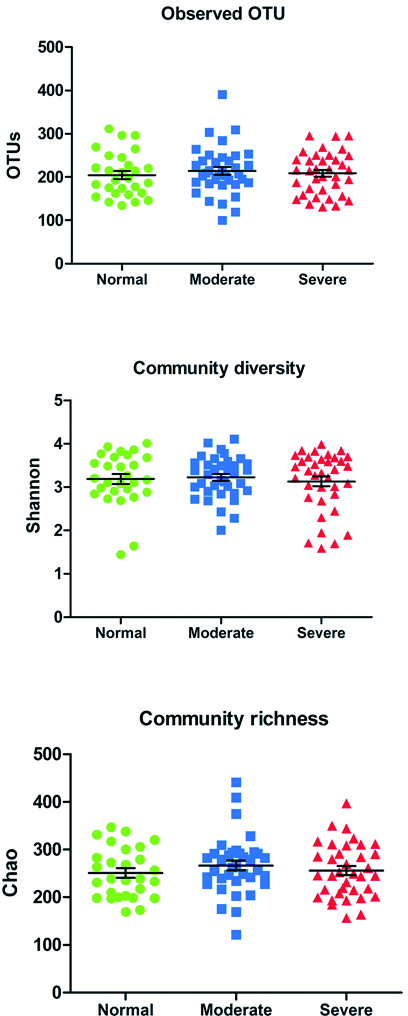

Supplement: Supplementary Figure S3 [file ijos201718x3.tif]
