# Supplementary material for: Marginal bone loss around non-submerged implants is associated with salivary microbiome during bone healing
Source: Int J Oral Sci. 2017 Jun 16;9(2):95–103. doi: 10.1038/ijos.2017.18 (PMC5518974; doi:10.1038/ijos.2017.18)
Supplement: Supplementary Table S1 [file ijos201718x4.docx]

| **Groups** | **Adonis** | **P-value** | **ANOSIM** | **P-value** |
| --- | --- | --- | --- | --- |
| N vs M | 0.012 | 0.668 | 0.019 | 0.797 |
| N vs S | 0.017 | 0.324 | 0.021 | 0.412 |
| M vs S | 0.013 | 0.46 | 0.004 | 0.770 |

N, normal group; M, moderate group; S, severe group.
