# Supplementary material for: Marginal bone loss around non-submerged implants is associated with salivary microbiome during bone healing
Source: Int J Oral Sci. 2017 Jun 16;9(2):95–103. doi: 10.1038/ijos.2017.18 (PMC5518974; doi:10.1038/ijos.2017.18)
Supplement: Supplementary Table S2 [file ijos201718x5.docx]

|  | **Species names or ID based on HOMD** | **Normal**  **(n=28)** | **Moderate**  **(n=36)** | **Severe**  **(n=36)** |
| --- | --- | --- | --- | --- |
| OTU10 | *Unclassified Rothia sp.* | 0.07±0.07 | 0.19±0.32 | 0.12±0.41 |
| OTU1000 | *Peptostreptococcus stomatis* | 0.68±0.71 | 0.39±0.45 | 0.41±0.59 |
| OTU101 | *Unclassified Desulfovibrio sp.* | 0.03±0.09 | 0.01±0.04 | 0.00±0.01 |
| **OTU103** | *Uncultured Lachnoanaerobaculum* | 0.14±0.16 | 0.08±0.10 | 0.07±0.10 |
| OTU109 | *Catonella sp. \| HOT_451 \| sp.* | 0.01±0.03 | 0.03±0.05 | 0.03±0.05 |
| OTU112 | *Uncultured Mobiluncus sp.* | 0.30±0.70 | 0.55±2.34 | 0.22±0.56 |
| OTU12 | *Uncultured Selenomonas sp.* | 0.87±1.21 | 2.31±4.83 | 1.78±2.77 |
| OTU121 | *Eubacterium infirmum* | 0.27±0.21 | 0.19±0.21 | 0.23±0.3 |
| OTU124 | *Chryseobacterium indoltheticum* | 0.02±0.06 | 0.01±0.02 | 0.01±0.01 |
| OTU126 | *Vibrio gigantis* | 0.24±0.58 | 0.23±0.31 | 0.14±0.21 |
| OTU129 | *Selenomonas sputigena* | 0.00±0.00 | 0.00±0.02 | 0.01±0.02 |
| OTU13 | *Bergeyella sp. \| HOT_900 \|* | 0.01±0.03 | 0.02±0.04 | 0.01±0.02 |
| OTU131 | *Neisseria oralis* | 0.28±0.39 | 0.45±1.16 | 0.67±1.39 |
| OTU132 | *Uncultured Brevibacillus sp.* | 0.02±0.12 | 0.01±0.04 | 0.00±0.01 |
| **OTU133** | *Eubacterium brachy* | 0.04±0.07 | 0.14±0.22 | 0.06±0.09 |
| OTU135 | *Uncultured Selenomonas sp.* | 0.41±0.85 | 0.48±0.85 | 0.65±1.21 |
| OTU139 | *Unclassified Corynebacterium sp.* | 0.01±0.04 | 0.03±0.15 | 0.00±0.01 |
| **OTU143** | *Uncultured Coprococcus sp.* | 0.01±0.02 | 0.02±0.04 | 0.05±0.12 |
| OTU144 | *Abiotrophia defectiva* | 0.02±0.04 | 0.13±0.30 | 0.05±0.10 |
| OTU147 | *Mitsuokella sp. \| HOT_131 \|* | 0.04±0.15 | 0.03±0.08 | 0.02±0.06 |
| OTU149 | *Prevotella salivae* | 0.51±0.68 | 0.32±0.62 | 0.41±0.61 |
| OTU150 | *Unclassified Tannerella sp.* | 0.01±0.02 | 0.00±0.01 | 0.02±0.03 |
| OTU155 | *Moraxella osloensis* | 0.01±0.01 | 0.02±0.03 | 0.01±0.01 |
| OTU156 | *Bacteroidetes [G-6] sp. \| HOT_516 \|* | 0.01±0.04 | 0.00±0.01 | 0.02±0.06 |
| OTU16 | *Uncultured Ruminococcaceceae sp.* | 0.50±0.74 | 1.33±3.82 | 1.77±3.99 |
| **OTU160** | *Treponema denticola* | 0.03±0.06 | 0.05±0.08 | 0.08±0.14 |
| OTU166 | *Cardiobacterium valvarum* | 0.00±0.01 | 0.01±0.04 | 0.00±0.00 |
| **OTU168** | *Streptococcus intermedius* | 0.12±0.17 | 0.28±0.35 | 0.42±1.09 |
| OTU174 | *Prevotella micans* | 0.02±0.04 | 0.01±0.02 | 0.04±0.10 |
| OTU175 | *Uncultured Saccharibacteria sp.* | 0.02±0.03 | 0.04±0.15 | 0.02±0.05 |
| OTU185 | *Eubacterium saphenum* | 0.08±0.18 | 0.11±0.21 | 0.14±0.26 |
| OTU187 | *Eubacterium nodatum* | 0.03±0.07 | 0.07±0.13 | 0.07±0.15 |
| OTU189 | *Kingella oralis* | 0.01±0.02 | 0.02±0.03 | 0.01±0.01 |
| OTU19 | *Dialister pneumosintes* | 0.01±0.02 | 0.02±0.08 | 0.02±0.04 |
| OTU192 | *Uncultured Uruburuella sp.* | 0.07±0.19 | 0.07±0.09 | 0.05±0.06 |
| OTU201 | *Uncultured Clostridiales sp.* | 0.01±0.02 | 0.01±0.02 | 0.01±0.02 |
| OTU202 | *SR1 [G-1] sp. \| HOT_345 \|* | 0.01±0.03 | 0.01±0.01 | 0.04±0.14 |
| OTU209 | *Unclassified Streptococcus sp.* | 1.42±1.46 | 1.97±2.35 | 1.57±2.60 |
| OTU217 | *Unclassified Anoxybacillus sp.* | 0.02±0.06 | 0.00±0.02 | 0.00±0.01 |
| OTU220 | *Prevotella saccharolytica* | 0.01±0.02 | 0.02±0.05 | 0.02±0.03 |
| OTU23 | *Unclassified Lautropia sp.* | 11.51±18.33 | 8.46±12.6 | 10.28±17.6 |
| OTU232 | *Moraxella lacunata* | 0.01±0.04 | 0.02±0.08 | 0.01±0.07 |
| **OTU233** | *Unclassified Streptococcus sp.* | 0.01±0.01 | 0.01±0.01 | 0.00±0.01 |
| OTU234 | *Uncultured Aggregatibacter sp.* | 0.28±0.78 | 0.26±0.40 | 0.21±0.56 |
| OTU239 | *Actinomyces odontolyticus* | 3.65±6.55 | 2.55±2.10 | 2.70±1.99 |
| OTU24 | *Prevotella shahi* | 0.13±0.14 | 0.15±0.19 | 0.11±0.16 |
| OTU244 | *Aeromonas hydrophila* | 0.07±0.19 | 0.07±0.09 | 0.04±0.05 |
| OTU248 | *Solobacterium moorei* | 0.04±0.06 | 0.05±0.08 | 0.03±0.07 |
| OTU251 | *Solobacterium moorei* | 0.41±0.52 | 0.34±0.55 | 0.47±0.99 |
| OTU26 | *Uncultured Rikenellaceae sp.* | 0.01±0.05 | 0.00±0.01 | 0.04±0.11 |
| OTU263 | *Unclassified Prevotellaceae sp.* | 0.03±0.05 | 0.05±0.10 | 0.02±0.07 |
| OTU269 | *Unclassified Microbacterium sp.* | 0.03±0.09 | 0.02±0.04 | 0.01±0.02 |
| OTU272 | *Unclassified Pasteurellacee sp.* | 0.10±0.24 | 0.10±0.15 | 0.06±0.10 |
| OTU276 | *Anaeroglobus geminatus* | 0.01±0.02 | 0.02±0.04 | 0.01±0.02 |
| OTU279 | *Prevotella pallens* | 1.11±1.58 | 0.76±1.49 | 0.68±1.37 |
| OTU284 | *Uncultured Lachnospiraceae sp.* | 0.12±0.17 | 0.14±0.25 | 0.27±0.62 |
| OTU286 | *Acinetobacter bohemicus* | 0.04±0.08 | 0.03±0.04 | 0.04±0.06 |
| OTU287 | *Pseudomonas cichorii* | 0.07±0.29 | 0.02±0.04 | 0.02±0.02 |
| **OTU288** | *Porphyromonas gingivalis* | 0.25±0.60 | 0.38±0.65 | 1.29±2.60 |
| OTU304 | *Unclassified Porphyromonas sp.* | 0.01±0.05 | 0.00±0.02 | 0.01±0.04 |
| **OTU306** | *Prevotella nanceiensis* | 0.32±0.31 | 0.20±0.32 | 0.19±0.23 |
| OTU308 | *Pseudomonas marincola* | 0.03±0.07 | 0.03±0.04 | 0.02±0.02 |
| OTU312 | *Unclassified Treponema sp.* | 0.02±0.06 | 0.03±0.09 | 0.02±0.05 |
| OTU315 | *Prevotella melaninogenica* | 4.65±4.78 | 3.31±5.03 | 3.18±4.57 |
| OTU320 | *Unclassified Rhizobium sp.* | 0.07±0.14 | 0.07±0.10 | 0.05±0.07 |
| OTU322 | *Uncultured Lautropia sp.* | 2.06±3.28 | 2.38±5.33 | 1.79±2.88 |
| OTU324 | *Propionibacterium acnes* | 0.02±0.03 | 0.03±0.06 | 0.01±0.03 |
| OTU328 | *Unclassified Marinomonas sp.* | 0.11±0.29 | 0.09±0.13 | 0.06±0.08 |
| OTU33 | *Brevundimonas diminuta* | 0.15±0.43 | 0.12±0.15 | 0.09±0.11 |
| OTU335 | *Unclassified Treponema sp.* | 0.01±0.02 | 0.03±0.09 | 0.01±0.03 |
| OTU340 | *Unclassified Acinetobacter sp.* | 0.02±0.05 | 0.02±0.05 | 0.01±0.02 |
| OTU341 | *Neisseria mucosa* | 0.43±0.70 | 1.20±1.82 | 2.23±6.09 |
| OTU345 | *Unclassified Acinetobacter sp.* | 0.05±0.09 | 0.04±0.05 | 0.03±0.13 |
| **OTU350** | *Rothia aeria* | 1.38±1.50 | 1.61±1.38 | 1.33±2.43 |
| OTU351 | *Unclassified Gracilibacteria sp.* | 0.04±0.08 | 0.02±0.07 | 0.06±0.14 |
| OTU355 | *Treponema sp. \| HOT_257 \|* | 0.02±0.04 | 0.11±0.23 | 0.06±0.12 |
| OTU359 | *Unclassified Desulfobulbus sp.* | 0.01±0.02 | 0.01±0.03 | 0.02±0.03 |
| OTU360 | *Alloprevotella sp. \| HOT_912 \|* | 0.01±0.01 | 0.02±0.04 | 0.06±0.16 |
| OTU364 | *Unclassified Acidovorax sp.* | 0.01±0.02 | 0.01±0.02 | 0.01±0.01 |
| OTU372 | *Treponema lecithinolyticum* | 0.01±0.02 | 0.01±0.03 | 0.01±0.03 |
| OTU373 | *Unclassified Ruminococcaceaae sp.* | 0.03±0.06 | 0.02±0.08 | 0.02±0.07 |
| OTU375 | *Unclassified Corynebacterium sp.* | 0.10±0.14 | 0.14±0.30 | 0.04±0.07 |
| OTU376 | *Corynebacterium argentoratense* | 0.00±0.01 | 0.17±0.70 | 0.04±0.13 |
| OTU377 | *Unclassified Thermus sp.* | 0.04±0.14 | 0.03±0.17 | 0.04±0.15 |
| OTU381 | *Unclassified Selenomonas sp.* | 1.50±2.71 | 1.91±3.38 | 2.61±3.9 |
| OTU382 | *Peptostreptococcaceae [XI][G-4] sp. \| HOT_369 \|* | 0.10±0.29 | 0.18±0.40 | 0.26±0.43 |
| OTU385 | *Unclassified Alloprevotella sp.* | 0.54±1.54 | 0.22±0.64 | 0.15±0.27 |
| OTU386 | *Cardiobacterium hominis* | 0.01±0.01 | 0.01±0.03 | 0.00±0.01 |
| OTU393 | *Capnocytophaga sputigena* | 0.29±0.76 | 0.33±0.68 | 0.59±1.45 |
| OTU397 | *Fusobacterium nucleatum subsp. vincentii* | 0.57±0.66 | 0.72±0.90 | 0.74±0.87 |
| OTU398 | *Unclassified Dialister sp.* | 0.05±0.07 | 0.07±0.09 | 0.07±0.20 |
| OTU400 | *Unclassified Capnocytophaga sp.* | 0.03±0.05 | 0.04±0.06 | 0.03±0.04 |
| OTU402 | *Prevotella enoeca* | 0.01±0.03 | 0.01±0.02 | 0.01±0.02 |
| OTU404 | *Streptococcus parasanguinis* | 3.42±2.72 | 4.31±3.71 | 3.33±2.62 |
| OTU407 | *Unclassified Oribacterium sp.* | 0.01±0.02 | 0.00±0.01 | 0.02±0.06 |
| OTU408 | *Leptotrichia hofstadii* | 0.10±0.13 | 0.15±0.29 | 0.10±0.13 |
| **OTU412** | *Unclassified Leptotrichia sp.* | 0.07±0.11 | 0.10±0.12 | 0.05±0.10 |
| **OTU414** | *Streptococcus agalactiae* | 0.04±0.05 | 0.07±0.08 | 0.05±0.07 |
| OTU423 | *Unclassified Leptotrichia sp.* | 0.01±0.03 | 0.03±0.07 | 0.01±0.03 |
| OTU424 | *Prevotella loescheii* | 0.01±0.02 | 0.02±0.04 | 0.04±0.11 |
| OTU430 | *Unclassified Parvimonas sp.* | 0.15±0.32 | 0.22±0.33 | 0.18±0.29 |
| OTU439 | *Treponema refringens* | 0.04±0.09 | 0.04±0.11 | 0.01±0.01 |
| OTU444 | *TM7 [G-1] sp. \| HOT_349 \|* | 0.02±0.05 | 0.08±0.31 | 0.03±0.06 |
| OTU447 | *Uncultured Stomatobaculum sp.* | 0.04±0.08 | 0.03±0.05 | 0.02±0.05 |
| **OTU448** | *Uncultured Alysiella sp.* | 0.02±0.07 | 0.01±0.01 | 0.00±0.01 |
| OTU45 | *Unclassified Capnocytophaga sp.* | 0.42±0.52 | 1.12±2.74 | 0.74±1.04 |
| OTU452 | *Gracilibacteria bacterium* | 0.20±0.66 | 0.05±0.17 | 0.09±0.26 |
| OTU456 | *Uncultured Bergeyalla sp.* | 0.01±0.05 | 0.02±0.09 | 0.03±0.16 |
| OTU458 | *Oribacterium asaccharolyticum* | 1.09±1.71 | 1.25±2.66 | 0.79±1.59 |
| OTU467 | *Uncultured Stomatobaculum sp.* | 0.20±0.23 | 0.11±0.17 | 0.17±0.21 |
| OTU473 | *Unclassified Treponema sp.* | 0.00±0.01 | 0.01±0.02 | 0.03±0.17 |
| **OTU477** | *Treponema sp. \| HOT_262 \|* | 0.01±0.03 | 0.03±0.05 | 0.13±0.67 |
| OTU478 | *Veillonella parvula* | 3.50±2.12 | 3.26±2.68 | 2.33±2.13 |
| OTU482 | *Prevotella denticola* | 0.02±0.05 | 0.07±0.13 | 0.06±0.15 |
| OTU488 | *Unclassified Neisseria sp.* | 0.01±0.03 | 0.03±0.07 | 0.01±0.01 |
| OTU489 | *Olsenella sp. \| HOT_807 \|* | 0.02±0.04 | 0.02±0.05 | 0.02±0.06 |
| OTU49 | *Uncultured Lautropia sp.* | 0.06±0.09 | 0.06±0.14 | 0.03±0.06 |
| OTU490 | *Tannerella forsythia* | 0.13±0.20 | 0.12±0.15 | 0.20±0.24 |
| OTU493 | *Uncultured Porphyromonas sp.* | 2.49±2.99 | 2.26±3.42 | 1.46±1.58 |
| OTU499 | *Prevotella sp. \| HOT_443 \|* | 0.07±0.17 | 0.05±0.11 | 0.07±0.15 |
| **OTU502** | *Unclassified Prevotella sp.* | 0.05±0.08 | 0.04±0.10 | 0.03±0.04 |
| **OTU503** | *Prevotella sp. \| HOT_304 \|* | 0.03±0.06 | 0.07±0.14 | 0.14±0.20 |
| OTU504 | *Actinomyces graevenitzii* | 1.02±1.79 | 0.53±0.88 | 1.34±2.90 |
| OTU513 | *Uncultured Oribacterium sp.* | 4.82±5.88 | 5.09±8.95 | 7.34±13.98 |
| OTU518 | *Uncultured Bergeyella sp.* | 0.01±0.05 | 0.01±0.02 | 0.03±0.13 |
| OTU519 | *Actinomyces marimammalium* | 0.02±0.05 | 0.02±0.02 | 0.01±0.02 |
| OTU522 | *Uncultured Selenomonas sp.* | 2.50±5.34 | 1.68±2.41 | 2.46±3.80 |
| OTU529 | *Unclassified Leptotrichia sp.* | 0.29±0.48 | 0.21±0.52 | 0.15±0.33 |
| OTU535 | *Stenotrophomonas maltophilia* | 0.02±0.04 | 0.01±0.02 | 0.01±0.01 |
| OTU538 | *Unclassified Leptotrichia sp.* | 0.03±0.06 | 0.01±0.02 | 0.01±0.03 |
| OTU547 | *Unclassified Saccharibacteria sp.* | 0.15±0.29 | 0.21±0.46 | 0.11±0.25 |
| OTU55 | *Treponema maltophilum* | 0.01±0.02 | 0.02±0.05 | 0.02±0.04 |
| OTU555 | *Parvimonas micra* | 0.04±0.09 | 0.03±0.07 | 0.11±0.32 |
| OTU556 | *Uncultured Bergeyella sp.* | 0.07±0.06 | 0.08±0.09 | 0.06±0.09 |
| OTU557 | *Uncultured Tenericutes sp.* | 0.12±0.18 | 0.20±0.48 | 0.17±0.24 |
| OTU558 | *Unclassified Lactococcus sp.* | 0.02±0.04 | 0.02±0.03 | 0.02±0.03 |
| OTU561 | *Lachnospiraceae [G-3] sp. \| HOT_100 \|* | 0.08±0.09 | 0.07±0.18 | 0.09±0.13 |
| OTU564 | *Capnocytophaga sp. \| HOT_878 \|* | 0.01±0.03 | 0.01±0.03 | 0.02±0.09 |
| OTU57 | *Unclassified Butyrivibrio sp.* | 0.76±1.60 | 0.76±1.46 | 0.49±1.19 |
| OTU570 | *Alloprevotella rava \| HOT_302 \|* | 0.07±0.12 | 0.05±0.10 | 0.12±0.39 |
| OTU574 | *Campylobacter showae* | 0.12±0.18 | 0.06±0.10 | 0.11±0.16 |
| OTU576 | *Haemophilus pittmaniae* | 0.06±0.08 | 0.05±0.05 | 0.10±0.25 |
| OTU577 | *Unclassified Cyanobacteria sp.* | 0.03±0.07 | 0.04±0.07 | 0.03±0.08 |
| OTU581 | *Unclassified Tenericutes sp.* | 0.04±0.07 | 0.05±0.16 | 0.05±0.13 |
| OTU583 | *Prevotella sp. \| HOT_306 \|* | 0.00±0.01 | 0.01±0.02 | 0.02±0.10 |
| **OTU587** | *Gemella morbillorum* | 1.02±0.80 | 1.40±1.34 | 0.84±1.00 |
| OTU591 | *Megasphaera micronuciformis* | 0.19±0.34 | 0.07±0.10 | 0.12±0.21 |
| OTU605 | *Mycoplasma faucium* | 0.01±0.02 | 0.02±0.04 | 0.04±0.1 |
| OTU607 | *Unclassified Prevotella sp.* | 0.02±0.05 | 0.03±0.08 | 0.02±0.03 |
| OTU609 | *Uncultured Ruminococcaceae sp.* | 0.29±0.38 | 0.30±0.39 | 0.37±0.43 |
| OTU613 | *Peptoniphilaceae [G-2] sp. \| HOT_790 \|* | 0.00±0.01 | 0.02±0.07 | 0.04±0.18 |
| OTU614 | *Raoultella planticola* | 0.07±0.20 | 0.06±0.10 | 0.03±0.05 |
| **OTU615** | *Haemophilus parainfluenzae* | 3.14±2.78 | 2.96±3.15 | 2.32±5.29 |
| OTU616 | *Selenomonas sputigena* | 0.01±0.01 | 0.02±0.04 | 0.02±0.05 |
| OTU621 | *Unclassified Leptotrichia sp.* | 0.22±0.83 | 0.05±0.14 | 0.02±0.05 |
| OTU626 | *Uncultured Neisseria sp.* | 7.03±5.85 | 7.12±8.23 | 7.63±10.37 |
| OTU629 | *Unclassified Pseudoalteromonas sp.* | 0.21±0.55 | 0.17±0.25 | 0.12±0.16 |
| OTU630 | *Unclassified Leptotrichia sp.* | 0.18±0.20 | 0.17±0.21 | 0.15±0.21 |
| **OTU631** | *Prevotella baroniae* | 0.01±0.01 | 0.01±0.03 | 0.08±0.17 |
| OTU632 | *Unclassified Leptotrichia sp.* | 0.96±2.03 | 0.40±0.54 | 0.33±0.47 |
| OTU64 | *Johnsonella sp. \| HOT_166 \|* | 0.03±0.08 | 0.02±0.05 | 0.02±0.04 |
| OTU644 | *Capnocytophaga granulosa* | 0.03±0.10 | 0.04±0.13 | 0.05±0.22 |
| OTU646 | *Prevotella sp. \| HOT_475 \|* | 0.02±0.04 | 0.01±0.03 | 0.05±0.13 |
| OTU65 | *Alloprevotella tannerae* | 0.17±0.29 | 0.09±0.16 | 0.24±0.82 |
| OTU650 | *Unclassified Streptococcus sp.* | 7.38±6.59 | 8.64±7.33 | 6.02±6.03 |
| OTU651 | *Fusobacterium periodonticum* | 1.62±1.52 | 0.92±0.93 | 1.60±2.38 |
| **OTU666** | *Unclassified Acholeplasma.sp* | 0.00±0.00 | 0.00±0.01 | 0.02±0.05 |
| **OTU668** | *Unclassified Lachnoanaerobaculum.sp* | 0.08±0.16 | 0.02±0.04 | 0.02±0.04 |
| OTU675 | *Prevotella oris* | 0.14±0.22 | 0.28±0.47 | 0.15±0.29 |
| OTU678 | *Syntrophomonadaceae [VIII][G-1] sp. \| HOT_435 \|* | 0.11±0.43 | 0.00±0.01 | 0.02±0.07 |
| OTU680 | *Streptococcus anginosus* | 0.05±0.10 | 0.06±0.08 | 0.04±0.05 |
| OTU684 | *Uncultured Firmicutes sp.* | 0.77±1.16 | 1.05±3.02 | 0.54±0.67 |
| OTU695 | *Mollicutes [G-1] sp. \| HOT_504 \|* | 0.00±0.02 | 0.01±0.05 | 0.00±0.01 |
| OTU700 | *Streptococcus mutans* | 0.04±0.17 | 0.05±0.16 | 0.01±0.03 |
| OTU703 | *Actinobaculum sp. \| HOT_848 \|* | 0.03±0.07 | 0.04±0.06 | 0.06±0.22 |
| **OTU704** | *Prevotella heparinolytica* | 0.01±0.05 | 0.01±0.03 | 0.07±0.18 |
| OTU71 | *Prevotella nigrescens* | 0.05±0.10 | 0.06±0.09 | 0.05±0.17 |
| OTU715 | *Peptostreptococcaceae [XI][G-7] sp. \| HOT_081 \|* | 0.01±0.03 | 0.02±0.05 | 0.03±0.06 |
| OTU717 | *Peptostreptococcaceae [XI][G-2] sp. \| HOT_091 \|* | 0.01±0.02 | 0.02±0.06 | 0.03±0.08 |
| OTU721 | *Uncultured Lachnoanaerobaculum sp.* | 0.01±0.01 | 0.01±0.01 | 0.02±0.04 |
| OTU726 | *Unclassified Actinomyces sp.* | 0.02±0.02 | 0.01±0.02 | 0.01±0.03 |
| OTU727 | *Unclassified Bacteroidales sp.* | 0.01±0.02 | 0.01±0.04 | 0.07±0.43 |
| OTU728 | *Uncultured Capnocytophaga sp.* | 0.03±0.09 | 0.07±0.23 | 0.04±0.15 |
| OTU732 | *Peptostreptococcaceae [XI][G-8] sp.*  *\| HOT_382 \|* | 0.00±0.01 | 0.01±0.02 | 0.01±0.05 |
| OTU734 | *Porphyromonas endodontalis* | 0.42±0.90 | 0.38±0.56 | 0.52±0.68 |
| **OTU737** | *Unclassified Peptoclostridium sp.* | 0.41±0.97 | 0.04±0.07 | 0.32±1.10 |
| OTU738 | *Unclassified Oribacterium sp.* | 0.00±0.00 | 0.01±0.03 | 0.01±0.01 |
| OTU746 | *Campylobacter concisus* | 0.42±0.52 | 0.35±0.65 | 0.48±1.06 |
| **OTU754** | *Unclassified Staphylococcus sp.* | 0.00±0.01 | 0.02±0.06 | 0.00±0.00 |
| OTU756 | *Pseudomonas orientalis* | 0.09±0.15 | 0.11±0.16 | 0.05±0.08 |
| OTU782 | *Haemophilus sputorum* | 0.01±0.01 | 0.01±0.03 | 0.00±0.01 |
| OTU790 | *Unclassified Neisseria sp.* | 0.03±0.05 | 0.06±0.15 | 0.10±0.33 |
| OTU791 | *Mogibacterium_diversum* | 0.24±0.23 | 0.17±0.20 | 0.21±0.27 |
| OTU80 | *Uncultured Anaerovorax sp.* | 0.06±0.31 | 0.01±0.03 | 0.02±0.07 |
| OTU804 | *Prevotella oulorum* | 0.10±0.22 | 0.13±0.34 | 0.13±0.4 |
| OTU809 | *Unclassified Porphyromonadaceae sp.* | 0.06±0.07 | 0.08±0.10 | 0.12±0.19 |
| OTU826 | *Actinomyces dentalis* | 0.01±0.01 | 0.01±0.01 | 0.01±0.03 |
| OTU829 | *Shuttleworthia satelles* | 0.01±0.03 | 0.03±0.06 | 0.01±0.02 |
| OTU830 | *Unclassified Fretibacterium sp.* | 0.06±0.13 | 0.12±0.27 | 0.22±0.49 |
| OTU832 | *Treponema sp. \| HOT_258 \|* | 0.01±0.14 | 0.03±0.10 | 0.02±0.04 |
| OTU846 | *Prevotella multisaccharivorax* | 0.01±0.02 | 0.03±0.08 | 0.02±0.06 |
| OTU847 | *Unclassified Proteiniphilum sp* | 0.01±0.04 | 0.01±0.03 | 0.03±0.10 |
| OTU848 | *Unclassified Acinetobacter sp.* | 0.04±0.09 | 0.03±0.05 | 0.03±0.03 |
| OTU85 | *Unclassified Rikenellaceae sp.* | 0.01±0.02 | 0.01±0.02 | 0.02±0.03 |
| OTU852 | *Unclassified Vibrio sp.* | 0.07±0.14 | 0.07±0.10 | 0.05±0.06 |
| OTU864 | *Uncultured Selenomonas sp.* | 0.10±0.13 | 0.42±1.65 | 0.19±0.36 |
| OTU866 | *Unclassified Prevotella sp.* | 0.02±0.02 | 0.02±0.04 | 0.03±0.05 |
| OTU871 | *Uncultured Bacteroidetes sp* | 0.00±0.00 | 0.01±0.03 | 0.02±0.08 |
| **OTU874** | *Prevotella aurantiaca* | 0.02±0.02 | 0.01±0.02 | 0.00±0.01 |
| **OTU880** | *Uncultured Actinomycetaceae sp.* | 0.04±0.06 | 0.03±0.04 | 0.01±0.01 |
| **OTU884** | *Unclassified Sphaerochaeta sp.* | 0.01±0.03 | 0.01±0.02 | 0.08±0.28 |
| OTU889 | *Unclassified Atopobium sp.* | 0.22±0.19 | 0.14±0.12 | 0.21±0.38 |
| OTU89 | *Prevotella sp. \| HOT_396 \|* | 0.16±0.42 | 0.14±0.12 | 0.12±0.21 |
| OTU893 | *Streptococcus salivarius* | 7.47±10.07 | 6.41±6.45 | 5.43±7.04 |
| **OTU894** | *Phocaeicola abscessus* | 0.02±0.03 | 0.02±0.03 | 0.06±0.12 |
| OTU897 | *Capnocytophaga granulosa* | 0.30±0.31 | 0.40±0.6 | 0.29±0.35 |
| OTU898 | *Actinomyces cardiffensis*  *\| HOT_850 \|* | 0.01±0.01 | 0.01±0.02 | 0.03±0.05 |
| OTU9 | *Prevotella sp. \| HOT_305 \|* | 0.02±0.05 | 0.02±0.06 | 0.01±0.04 |
| OTU905 | *Unclassified Delftia sp.* | 0.02±0.07 | 0.02±0.03 | 0.01±0.02 |
| OTU907 | *Unclassified Chryseobacterium sp.* | 0.03±0.08 | 0.02±0.04 | 0.02±0.02 |
| OTU909 | *Unclassified Propionibacterium sp.* | 0.04±0.06 | 0.05±0.12 | 0.02±0.03 |
| OTU91 | *Unclassified Peptococcus sp.* | 0.08±0.11 | 0.06±0.09 | 0.04±0.05 |
| OTU910 | *Treponema socranskii* | 0.03±0.04 | 0.06±0.13 | 0.09±0.29 |
| OTU911 | *Mycoplasma salivarium* | 0.01±0.01 | 0.03±0.12 | 0.02±0.06 |
| OTU913 | *Filifactor alocis* | 0.12±0.19 | 0.21±0.28 | 0.27±0.37 |
| OTU918 | *Unclassified Actinomyces sp.* | 0.03±0.04 | 0.03±0.07 | 0.11±0.40 |
| OTU92 | *Unclassified Rikenellaceae sp.* | 0.01±0.02 | 0.02±0.07 | 0.04±0.10 |
| OTU920 | *Eubacterium yurii* | 0.45±1.02 | 0.31±0.65 | 0.37±0.43 |
| OTU927 | *Prevotella pleuritidis* | 0.01±0.02 | 0.01±0.02 | 0.02±0.05 |
| OTU928 | *Eggerthia catenaformis* | 0.01±0.02 | 0.02±0.05 | 0.01±0.01 |
| OTU930 | *Unclassified Capnocytophaga sp.* | 0.08±0.18 | 0.03±0.06 | 0.30±0.90 |
| OTU932 | *Uncultured Lachnoanaerobaculum sp.* | 0.26±0.26 | 0.20±0.20 | 0.26±0.32 |
| OTU935 | *Catonella morbi* | 0.79±0.73 | 0.91±0.99 | 0.81±1.08 |
| OTU938 | *Uncultured Treponema sp.* | 0.01±0.02 | 0.06±0.14 | 0.09±0.41 |
| OTU942 | *Unclassified Clostridiales sp.* | 0.09±0.18 | 0.19±0.49 | 0.13±0.29 |
| OTU945 | *Uncultured Fretibacterium sp.* | 0.17±0.28 | 0.48±1.19 | 0.58±0.92 |
| OTU948 | *Granulicatella adiaces* | 1.22±0.99 | 1.69±1.37 | 1.29±1.79 |
| **OTU952** | *Uncultured Alloprevotella sp.* | 0.26±0.67 | 0.05±0.09 | 0.06±0.09 |
| OTU960 | *Treponema medium* | 0.05±0.07 | 0.10±0.15 | 0.09±0.17 |
| OTU963 | *Bacillus methylotrophicus* | 0.00±0.01 | 0.03±0.06 | 0.01±0.02 |
| OTU970 | *Neisseria elongata* | 0.34±0.34 | 0.48±0.60 | 0.35±0.59 |
| OTU974 | *Unclassified Sphingomonas sp.* | 0.03±0.07 | 0.03±0.06 | 0.02±0.04 |
| OTU979 | *Corynebacterium durum* | 0.25±0.43 | 0.28±0.43 | 0.25±0.63 |
| OTU980 | *Unclassified Veillonella sp.* | 0.01±0.02 | 0.00±0.01 | 0.01±0.03 |
| OTU981 | *Unclassified Butyrivibrio sp* | 0.05±0.15 | 0.02±0.04 | 0.04±0.09 |
| OTU982 | *Uncultured Bacteroidetes sp.* | 0.01±0.02 | 0.01±0.02 | 0.01±0.04 |
| OTU988 | *Bergeyella sp. \| HOT_319 \|* | 0.02±0.07 | 0.01±0.02 | 0.01±0.01 |
| OTU99 | *Leptotrichia sp. \| HOT_212 \|* | 0.13±0.15 | 0.06±0.09 | 0.12±0.16 |
| **OTU997** | *Unclassified Actinomyces sp.* | 0.44±0.54 | 0.65±1.37 | 0.38±0.84 |
| OTU999 | *TM7 [G-3] sp. \| HOT_351 \|* | 0.23±0.51 | 0.19±0.34 | 0.37±1.47 |
